# Type-specific photoreceptor loss in pigeons after disruption of parasympathetic control of choroidal blood flow by the medial subdivision of the nucleus of Edinger–Westphal

**DOI:** 10.1017/S0952523816000043

**Published:** 2016-05-04

**Authors:** A. REINER, T.T. WONG, C.C. NAZOR, N. DEL MAR, M.E.C. FITZGERALD

**Affiliations:** 1 Department of Anatomy & Neurobiology, The University of Tennessee Health Science Center, Memphis, Tennessee; 2 Department of Ophthalmology, The University of Tennessee Health Science Center, Memphis, Tennessee; 3 Department of Biology, Christian Brothers University, Memphis, Tennessee

**Keywords:** Choroidal blood flow, Parasympathetic regulation, Photoreceptors, Pigeon, Light damage

## Abstract

The medial part of the nucleus of Edinger–Westphal (EWM) in birds mediates light-regulated adaptive increases in choroidal blood flow (ChBF). We sought to characterize the effect of loss of EWM-mediated ChBF regulation on photoreceptor health in pigeons housed in either moderate intensity diurnal or constant light (CL). Photoreceptor abundance following complete EWM destruction was compared to that following a lesion in the pupil control circuit (as a control for spread of EWM lesions to the nearby pupil-controlling lateral EW) or following no EW damage. Birds were housed post-lesion in a 12 h 400 lux light/12 h dark light cycle for up to 16.5 months, or in constant 400 lux light for up to 3 weeks. Paraformaldehyde–glutaraldehyde fixed eyes were embedded in plastic, sectioned, slide-mounted, and stained with toluidine blue/azure II. Blinded analysis of photoreceptor outer segment abundance was performed, with outer segment types distinguished by oil droplet tint and laminar position. Brains were examined histologically to assess lesion accuracy. Disruption of pupil control had no adverse effect on photoreceptor outer segment abundance in either diurnal light or CL, but EWM destruction led to 50–60% loss of blue/violet cone outer segments in both light conditions, and a 42% loss of principal cone outer segments in CL. The findings indicate that adaptive regulation of ChBF by the EWM circuit plays a role in maintaining photoreceptor health and mitigates the harmful effect of light on photoreceptors, especially short wavelength-sensitive cone photoreceptors.

## Introduction

**Fig. 1.**
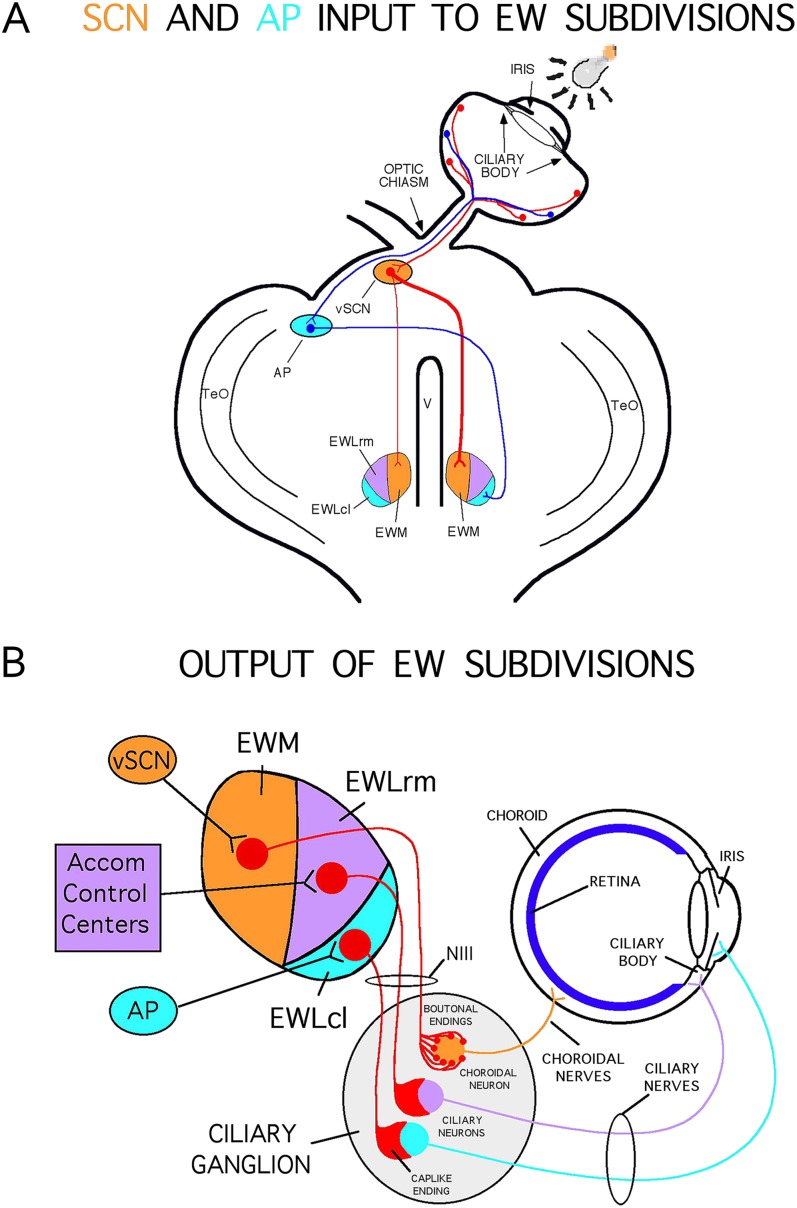
Schematized horizontal views of midbrain and eye in pigeon showing the central (**A**) and peripheral (**B**) circuitry for the visual pathways to the nucleus of Edinger–Westphal (EW) that drive ChBF increases and pupil constriction. (**A**) The pathway shown with red lines depicts the crossed projection from the retina to the vSCN that, in turn, has a mainly contralateral projection to EWM, which controls ChBF *via* its ipsilateral projection to choroidal neurons of the CG, as depicted in (**B**). The pathway depicted with blue lines in (**A**) shows a crossed projection from the retina to AP, which then projects to the contralateral EWLcl, which controls the pupillary light reflex (PLR) *via* an ipsilateral projection to pupilloconstrictive neurons of the CG, as depicted in (**B**). (**B**) The peripheral circuitry controlling ChBF and PLR, with EW, the CG, and the eye all drawn in horizontal view. The subdivisions of EW project ipsilaterally *via* the oculomotor nerve to the CG, where the projection from EWM terminates on choroidal neurons that project to choroidal blood vessels. Projections from both the rostromedial part of lateral EW and from EWLcl terminate on ciliary neurons that project to the ciliary body and the iris, and control accommodation and the PLR, respectively. The subdivisions of EW are color-coded in (**A** and **B**), and the projections of each to the eye *via* the CG in (**B**) are as well. Other abbreviations: lateral subdivision of the nucleus of Edinger–Westphal (EWL); lateral reticular formation (LRF); optic tectum (TeO).

The choroidal blood supply to the outer retina is essential for the health of photoreceptors (Bill, 1984; Yancey & Linsenmeier, 1988, 1989). Choroidal vessels are innervated by parasympathetic, sympathetic, and sensory nerve fibers, which regulate choroidal vessel dilation and thereby choroidal blood flow (ChBF) (Bill, 1984; Stone et al., 1987; Cuthbertson et al., 1996, 1997, 2003; Reiner et al., 2012). We have previously described the central and peripheral components of the neural pathway in birds by which retinal information can reflexively increase ChBF (Gamlin et al., 1982; Fitzgerald et al., 1990*b*, 1996, 2001; Reiner et al., 1991; Cuthbertson et al., 1996). This neural circuit arises from retinal ganglion cells (Fig. 1) that project to the contralateral visual suprachiasmatic nucleus (vSCN), which itself then projects to the medial part of the nucleus of Edinger–Westphal (EWM), mainly on the same side as the eye of origin of the circuit (Cantwell & Cassone, 2006*a*,*b*). The EWM in turn projects to the ipsilateral ciliary ganglion (CG), where it terminates as boutonal endings on choroidal neurons of the CG, which innervate choroidal blood vessels (Gamlin et al., 1982; Reiner et al., 1983; Reiner et al., 1991; Cuthbertson et al., 1996). The CG terminals in the choroid produce vasodilation using muscarinic-endothelial nitric oxide mechanisms (Zagvazdin et al., 1996, 2000). This circuit is activated by retinal illumination (Fitzgerald et al., 1996), and it may thus serve to match ChBF to retinal activity-dependent need.

EWM lesions or choroidal nerve transections reduce basal ChBF in birds (Shih et al., 1994; Fitzgerald et al., 1996), and prevent light-mediated reflexive increases in ChBF (Fitzgerald et al., 1996), thereby likely causing chronic choroidal insufficiency. Consistent with this premise, we have found that destruction of EWM in young adult pigeons maintained in normal diurnal light (DL) greatly increases Müller cell expression of glial fibrillary acidic protein (GFAP) throughout the entire depth and topographic extent of the ipsilateral retina, up to a year post-lesion (Fitzgerald et al., 1990*a*; Kimble et al., 2006). Moreover, EWM lesions in young adult pigeons reduce visual acuity at about one year after the lesions (Hodos et al., 1998). Thus, our findings support the view that impairment of parasympathetic control of ChBF may harm the retina. Age-related decline in parasympathetic choroidal innervation has been observed in humans as well as in pigeons, in association with declines in basal ChBF (Grunwald et al., 1998; Fitzgerald et al., 2001, 2005; Ito et al., 2001; Jablonski et al., 2007). The precise consequences of impaired parasympathetic control of ChBF for the health of any particular retinal cell type have not, however, been established. Because of their critical dependence on ChBF, we conducted blinded quantitative analysis of photoreceptor outer segment abundance in pigeon retina following electrolytic destruction of EWM. Since it seemed possible that any protective benefit of ChBF regulation by EWM for photoreceptor health might be especially manifested under more stressful lighting conditions, we studied pigeons housed in both normal 400 lux 12 h light to 12 h dark cycle, as well as pigeons housed in constant 400 lux light. The results show that short wavelength (blue/violet) cones and principal cones are particularly vulnerable to ChBF deficiency caused by disrupted parasympathetic choroidal regulation, and constant light (CL) accentuates the vulnerability.

## Materials and methods

### Subjects

Fifty-three male and female White Carneaux pigeons (400–600 g), obtained from the Palmetto Pigeon Farm, the University of Maryland, or Duke University, were used. All animal studies were performed in accordance with a protocol approved by the Institutional Animal Care and Use Committee of the University of Tennessee Health Science Center (UTHSC) and complied with the National Institutes of Health and Society for Neuroscience guidelines, and the ARVO statement on the Use of Animals in Ophthalmic and Vision Research. Prior to the study, birds were maintained on a 12 h 400 lux light to 12 h dark photoperiod (12L–12D) in a fly cage, and had food and water access *ad libitum*. Forty-three of the animals received an electrolytic lesion that targeted either EWM or area pretectalis (AP). Ten pigeons received either a sham lesion or no surgery, and served as control birds. The EWM lesions were made to disrupt parasympathetic control of ChBF by the EWM circuit shown in Fig. 1. The AP lesions served as a control for the inadvertent but generally unavoidable effects of EWM lesions on the lateral pupil control part of EW (i.e., EWL), specifically the caudolateral part of EWL (EWLcl) that controls pupil constriction (Reiner et al., 1983; Gamlin et al., 1984). In principle, damage to the EWLcl that causes a fixed, dilated pupil could by itself result in light-mediated damage to the retina (Li et al., 1995; de Raad et al., 1996). To assess the effects of a fixed dilated pupil on the retina, the AP, which is the retinorecipient pretectal source of visual input to the EWLcl (Fig. 1) and the homologue of the mammalian olivary pretectal nucleus, was unilaterally destroyed in some pigeons (Reiner et al., 1983; Gamlin et al., 1984). Since AP receives luminance-related retinal input from the contralateral eye and in turn projects contralaterally to the pre-pupillomotor neurons of the EWLcl, AP mediates the pupillary light reflex for the contralateral eye and provides tonic drive to the caudolateral part of the contralateral EWL (Reiner et al., 1983; Gamlin et al., 1984). The destruction of AP, consequently, dilates the pupil and eliminates the pupillary light reflex in the contralateral eye, as does a lesion of the EWLcl ipsilateral to that eye.

### Lesions of the nucleus of EW or AP

For electrolytic lesions, animals were deeply anesthetized with ketamine (66 mg/kg) and xylazine (6.6 mg/kg), and secured in a stereotaxic device. Pigeons ranged from 0.5–3 years of age at the time of lesion. The EW and AP were targeted using coordinates from the stereotaxic atlas of the pigeon brain of Karten & Hodos (1967). Body temperature was maintained using a Harvard heating blanket. Electrode placement in EW or AP was confirmed by monitoring the effects of electrical activation (100 Hz, 0.5 ms pulse duration, 40–100 *µ*A pulse amplitude) *via* an insulated stainless steel electrode (AM Systems, Carlsborg, WA) on the ipsilateral pupil in the case of EW activation, and contralateral pupil in the case of AP activation. Upon electrode placement that yielded low threshold brisk pupil constriction, 1 mA constant anodal current was passed through the electrode for 30 seconds to destroy EW or AP. In a few cases, bilateral lesions were made. Animals were monitored for 24 h post surgery, and then placed in individual cages until fully recovered from surgery and anesthetic (postoperative analgesic was given). The birds were housed subsequently in either cyclic 12 h 400 lux to 12 h dark (12L/12D) for up to 16.5 months or in constant 400 lux for up to three weeks, using fluorescent lighting. Birds surviving for more than 4 months in DL were, in some cases, moved to fly cages with fluorescent lighting after individual cage housing. The abbreviation DL is used for the 12L–12D condition, and CL for the 24L–0D condition.

### Histology and immunohistochemistry—brain

The birds were deeply anesthetized and transcardially perfused with fixative consisting of 4% paraformaldehyde–0.1 m lysine–0.01 m sodium periodate in 0.1 m sodium phosphate buffer (pH 7.4). After perfusion, the brains were removed, cryoprotected in 20% sucrose–10% glycerol in 0.1 m sodium phosphate buffer, and sectioned at 40 *µ*m in six series. One series was mounted on slides as brains were sectioned frozen on a sliding microtome, and stained with 0.2% cresyl violet to determine lesion accuracy. If needed to determine if the lesion damaged all or only part of EW, additional series of sections were processed for immunocytochemistry using an antibody against choline acetyltransferase (ChAT) to detect EW neurons, which are cholinergic (Reiner et al., 1991). To determine if EWM or its input from the vSCN was destroyed, immunolabeling for substance P (SP), which is enriched in terminals of the vSCN input to EWM and thus delineates EWM and its input from the vSCN (Gamlin et al., 1982), was also carried out on one or more series from animals in which the lesion was in the region of the tract from the vSCN to EWM.

### Histology—retina

After transcardial perfusion, eyes were also removed from the head, the corneas cut away, and the eyecups transferred to an electron microscopy grade fixative consisting of 2% gluteraldehyde–2% paraformaldehyde–0.05% acrolein in a 0.1 m sodium cacodylate buffer. The eyecups were stored in this solution until processed for plastic embedding, at which time they were divided into four quadrants (superior, temporal, nasal, and inferior), with the red field occupying the superior quadrant (Fitzgerald et al. 2001). These quadrants were further cut into 2–3 separate wedges each for the central and peripheral retina, and rinsed in 0.1 m sodium cacodylate 3–5 times, immersed in 1% osmium tetroxide solution (in 0.1 m sodium cacodylate), dehydrated in an ascending series of alcohols, infiltrated in an increasing percentage of epoxy resin, and embedded in plastic molds. One-half to one-micron sections were obtained from blocks containing superior central retina using an Ultracut E (Reichert, Vienna, Austria), and stained with toludine blue/azure II solution. All analyses were conducted on the superior central retina, as in Fitzgerald et al. (2001), since it is the high acuity area in pigeon retina (Hodos et al., 1991).

### Classification into lesion groups

**Fig. 2.**
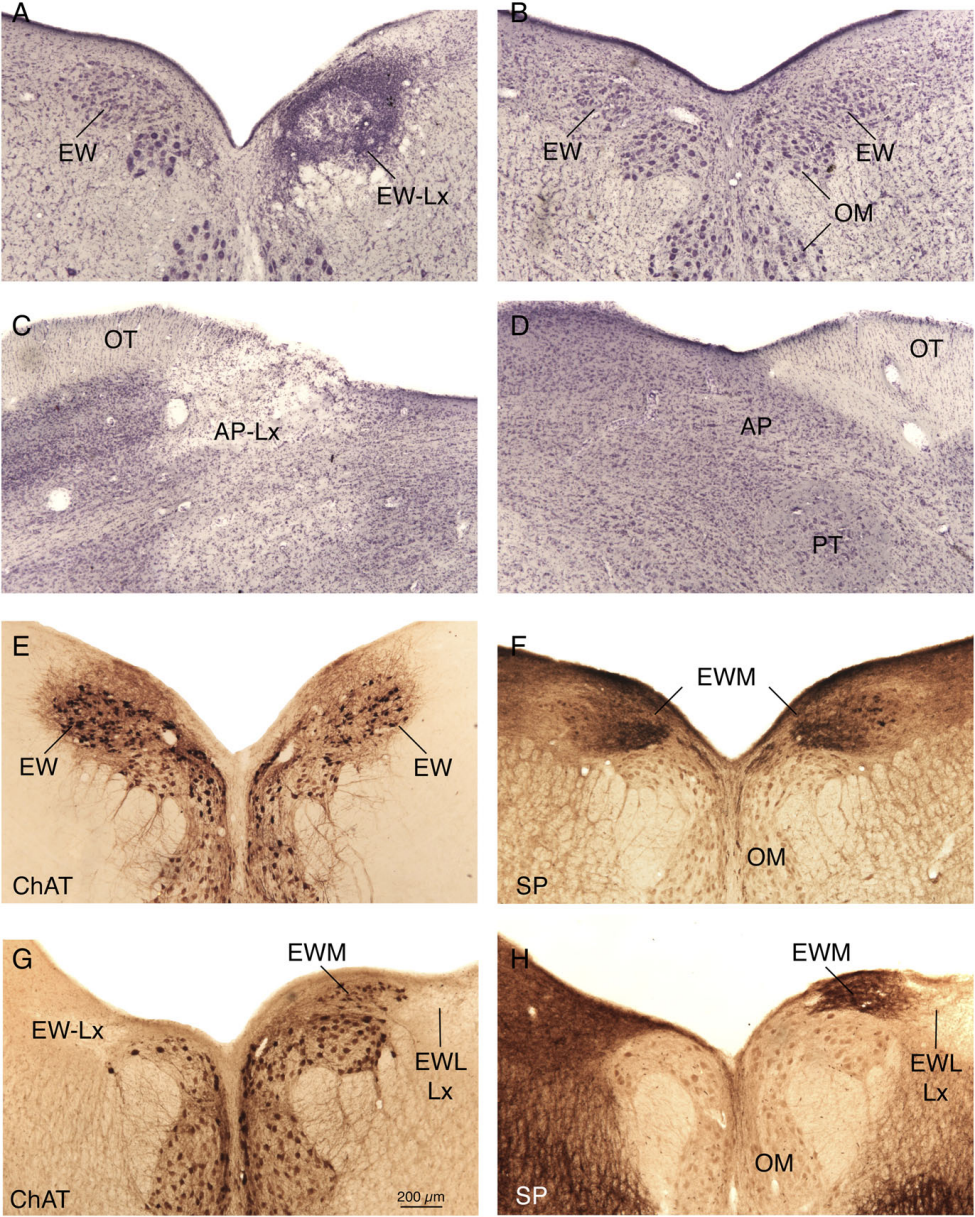
Images showing examples of lesions used to classify pigeons into groups. Image (**A**) shows a complete right EW lesion (EW-Lx) in a bird housed in 12L/12D DL, and for comparison image (**B**) shows normal EW on both sides of the brain from a bird that received a left AP lesion and was subsequently housed in 12L/12D DL. Images (**C** and **D**) show the left lesioned AP (AP-Lx) and right unlesioned AP, respectively, from the same bird as shown in (**B**). The lesions in (**A** and **C**) resulted in a fixed dilated pupil in the right eye, and a loss of the pupil light reflex. The images in E and F show sections immunostained for ChAT and SP, respectively, from a pigeon that received a left AP lesion. The images show that SP+ fibers, which arise from the vSCN, terminate in medial EW. Images (**G** and **H**) show immunostained sections for ChAT and SP, respectively, from a pigeon that received a bilateral EW lesion and was subsequently housed in 12L/12D DL. In this case, the immunostaining revealed a complete lesion on the left but substantial sparing of the right EWM and its input from the vSCN. Due to the complete destruction of the right EWL (with concomitant loss of the pupil light reflex) and the substantial sparing of the right EWM and its input from vSCN, the right eye for this bird was classified as AP-like. The magnification is the same in all images.

Brain histological outcome and pupil light reflex assessment were used to categorize animals into lesion outcome groups (Fig. 2). Birds with lesions targeting EW or AP were categorized as a lesion miss if EW or AP was undamaged, if the lesion was without effect on the pupil light reflex, and if the lesion did not affect suprachiasmatic nucleus input to EWM (Gamlin et al., 1982). Based on our analysis, birds were classified into three groups: (1) control birds that either had no surgery (nine birds), had a sham lesion (one bird), or had a lesion that missed the intended EW or AP target and had no effect on the pupil light reflex or input to EWM (two birds); (2) AP or EWLcl lesion birds that sustained an AP lesion (13 birds), or EW damage that only impaired the pupillary light reflex (i.e., destruction of EWLcl with less than 50% involvement of EWM) (six birds); and (3) EWM lesion birds that sustained greater than 90% EWM destruction irrespective of encroachment on EWLcl (22 birds). For the birds housed in 12L–12D lighting, there were 12 birds in the control category, 14 birds in the AP/EWLcl (eight AP and six EWLcl) category, and 15 in the EWM category. In the case of the AP lesions (all to the left AP), the contralateral eye (i.e., right) was used in analysis and is termed the experimental affected eye, while in the case of EW lesions, the ipsilateral eye was used for analysis and is termed the experimental affected eye. In the case of control eyes from birds housed in 12L–12D lighting, both eyes were used in the analysis for four birds. In these cases, data for the two eyes were averaged together per bird and used in analysis of group effects. In the text, table and graphs, we will refer to this group as control-DL. We did not use eyes contralateral to EWM lesions as controls because our prior study showed that EWM destruction slightly affects the contralateral eye (Kimble et al., 2006). Three EWM birds among those housed in DL had bilateral EWM lesions. In these cases as well, data for the two eyes were averaged together and used in analysis of group effects. In the text, table and graphs, we will refer to the birds with EWM destruction housed in DL as the EWM-DL group. In the case of those birds with AP or EWLcl lesions housed in DL, we use the term AP-DL for simplicity, and because the EWLcl lesions are AP-like.

For the CL condition, there were five birds in the AP category and seven in the EWM category. In the case of the AP lesions (all to left AP), the contralateral eye (i.e., right) was again the experimentally affected eye used in analysis, while in the case of EWM lesions, the affected ipsilateral eye was again used in the analysis. These groups will be referred to as AP-CL and EWM-CL, respectively. As we had no CL birds without either AP or EWM lesions, we used the eyes ipsilateral to the AP lesion (i.e., the left eye) as the control eyes. We expected this to serve effectively as a control eye since pupil control in pigeons is entirely crossed, and there was no pupil impairment in eyes ipsilateral to AP lesions. Moreover, we found no statistically significant differences between the left (i.e., unaffected) eyes of AP birds housed in 12L–12D lighting and control birds housed in 12L–12D lighting for any of our endpoints. We refer below to the left control eyes of the AP-CL birds as the control-CL group, for symmetry to the control-DL group. A summary of these groups is presented in Table 1.

**Table 1.** Summary of the groups, showing the number of birds per group, and the ages and survival times for the animals whose eyes are used in these groups.

Group	Number of pigeons	Mean survival postlesion in months	Shortest survival postlesion in months	Longest survival postlesion in months	Mean age at sacrifice in months	Youngest age at sacrifice in months	Oldest age at sacrifice in months
Control-DL	12	6.59	0.50	12.50	26.3	9.5	60
AP-DL	14	8.23	0.50	16.50	26.8	7.5	54
EWM-DL	15	5.60	0.25	16.25	16.7	8.5	30
Control-CL	5	0.45	0.25	0.75	10.3	9.5	10.8
AP-CL	5	0.45	0.25	0.75	10.3	8.25	11.8
EWM-CL	7	0.46	0.25	0.75	10.5	9.5	10.8

### Photoreceptor analysis

**Fig. 3.**
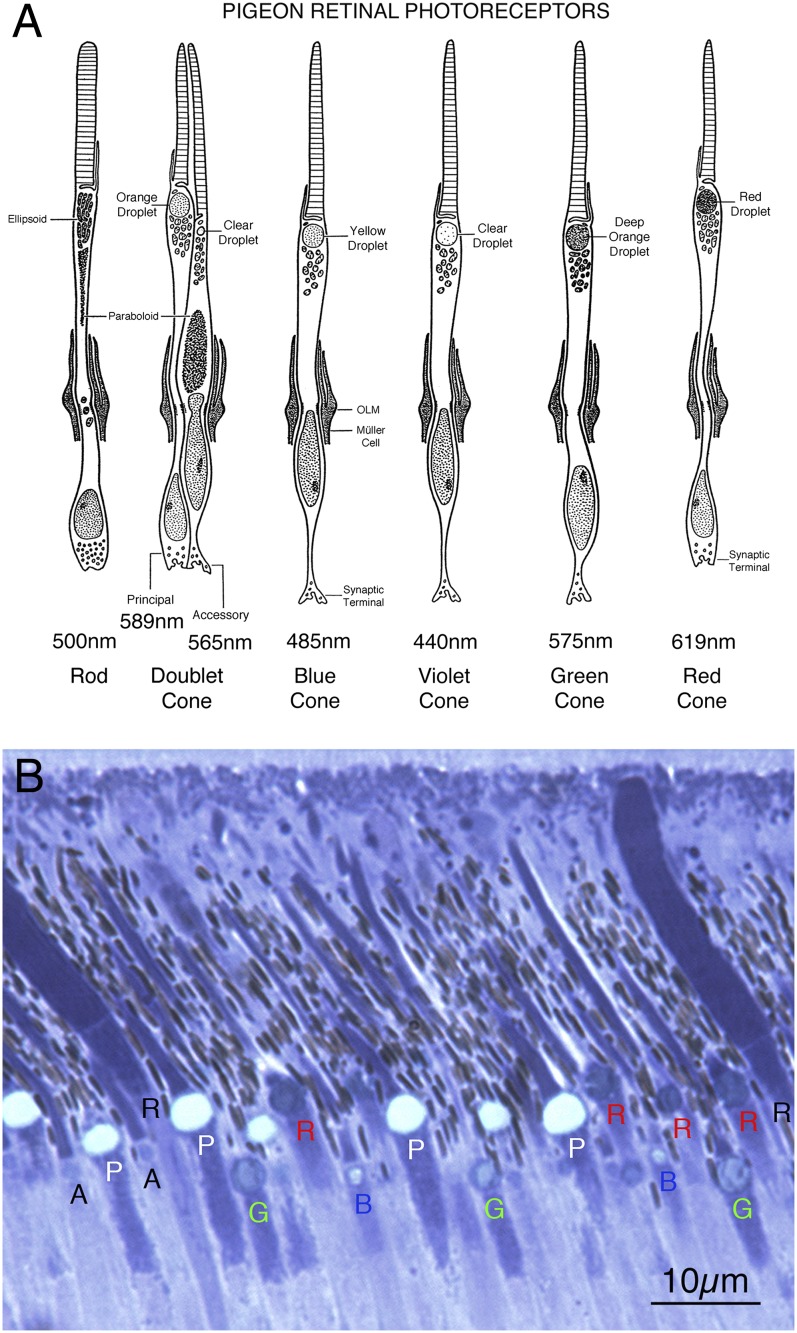
Schematic (**A**) illustrating the six different cone photoreceptor types as defined by their lipid droplet–photopigment combination, and the one type of rod photoreceptor present in the pigeon retina, as identified and characterized in prior studies noted in the text. Image (**B**) shows retinal photoreceptor outer segments in normal pigeon retina. Red cones (red R), principal cones (white P), green cones (green G), violet/blue cones (blue B), and rods (black R) are identifiable in B by the traits shown in (**A**). Accessory cones (black A) are also evident in (**B**). Schematic (**A**) is adapted and modified for pigeon from Fig. 1 of Morris and Shorey (1967).

The pigeon retina contains six different cone photoreceptor types as defined by their lipid droplet–photopigment combination and one type of rod photoreceptor (Fig. 3) (Mariani & Leurre-Dupree, 1978; Bowmaker et al., 1997). Cone oil droplets serve as narrow long-pass filters that together with the cone photopigment determine the spectral responses of the cone type (Bowmaker et al., 1997; Vorobyev, 2003; Hart & Vorobyev, 2005). In our sections, although oil droplet color was not discernible, the size and darkness of the oil droplet, and its relative location in the inner or outer row of oil droplets served to distinguish photoreceptor outer segment types. Of the six cone types, two always occur as a gap junction-coupled pair termed the double cone, whose individual members are the principal cone and accessory cone (Smith et al., 1985; Bowmaker et al., 1997). We recognized principal cones by the location of their large, pale oil droplet in the outer row of oil droplets. The accessory cone possesses a very narrow outer segment with a small circular entity below the outer segment that some have called a clear oil droplet (Meyer & Cooper, 1966; Meyer & May, 1973; Mariani & Leurre-Dupree, 1978; López-López et al., 2008), and some have not (Morris & Shorey, 1967; Bowmaker et al., 1997; Hart et al., 1999; Kram et al., 2010). Because of its narrow outer segment, an accessory cone is not always evident in the same plane of section as its associated principal cone. Due to their thin outer segment and indistinct droplets, we did not count accessory cones. Among the remaining four single cone types, red cones have a large dark red oil droplet in the outer row of oil droplets, and green cones have a large, dark green oil droplet in the inner row of oil droplets. Thus, the outer segments of these two cone types can be readily distinguished by the size and laminar position of their oil droplets. The blue and violet cones, however, possess moderately sized, pale oil droplets in the inner oil droplet row (Vorobyev, 2003). Thus, although we could identify blue cone and violet cone outer segments, we could not distinguish between them, and thus treat them here as one class. Finally, rod outer segments are easily recognized by their wide and untapered shape and lack of an oil droplet.

Based on these characteristics, we quantified the outer segment abundance of principal cones, blue–violet cones, green cones, red cones, and rods. Images of the outer retina were captured at high magnification (400×), with at least 1 mm of retina photographed for each eye and each animal. Blinded analysis was then conducted on coded images of the abundance of the outer segments of the different photoreceptor types. To avoid planar counting artifacts, we only counted photoreceptors with evident outer segments just above the oil droplet, and only counted rods traversing the oil droplet rows. Thus, our measure of photoreceptor abundance is not an oil droplet or outer segment count *per se* and may provide a lower estimate of photoreceptor abundance per length of retina than cell body, oil droplet, or outer segment counts of photoreceptor abundance do (Meyer & May, 1973; Bowmaker et al., 1997; Kram et al., 2010). Our goal was, however, to detect relative changes in specific photoreceptor types between groups as a function of lesion or lighting condition, rather than provide photoreceptor type counts per unit of retina *per se*. Photoreceptor abundance is presented per 100 *µ*m length of retina sampled.

### Statistical analysis

The effects of post-lesion survival time on photoreceptor abundance for each type were assessed by regression analysis for DL-housed birds, and CL-housed birds. For birds sustaining no lesion, the time spent in housing since arrival at UTHSC was regarded as the survival time. The regression analysis in general showed an effect of post-lesion survival time on abundance per photoreceptor type in only a limited number of cases. Any significant effects revealed by regression analysis are described below. In light of the limited effects of post-lesion survival time on photoreceptor abundance, as the main approach for determining the effects of the lesion manipulations or lighting conditions, we analyzed effects across groups using mixed-model 2-way Analysis of Variance (ANOVA), performed using Statistical Analysis Software (SAS), with three lesion levels (control, AP and EWM) and two lighting levels (diurnal *versus* constant), with individual comparisons between groups assessed by Fischer PLSD. Our approach for assessing effects of lesion and/or lighting condition was to compare each experimental condition to the control-DL condition to determine if it was significantly different. Because of the large number of comparisons, to limit false detection of differences (type 1 error), we set the significance level at 0.0125. Results are presented as mean ± SEM (Standard Error of the Mean).

## Results

### Principal cone outer segments

**Fig. 4.**
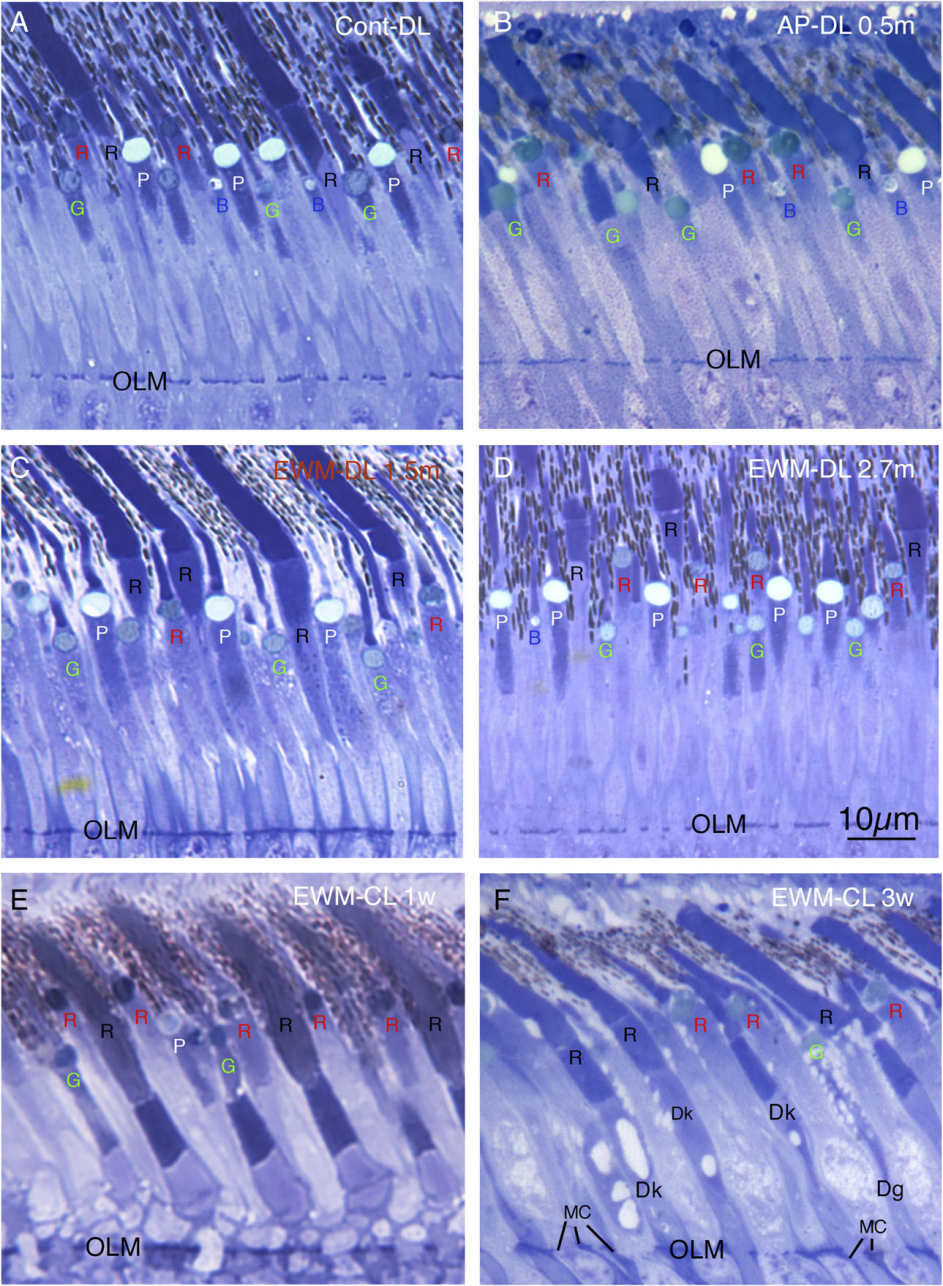
Images showing photoreceptor outer and inner segments in an eye unaffected by any lesion (**A**), in an eye affected by a contralateral AP lesion from a bird housed in normal DL for 0.5 months (**B**), in an eye affected by EWM destruction from a bird housed in normal DL for 1.5 months (**C**), in an eye affected by EWM destruction from a bird housed in normal DL for 2.7 months (**D**), in an eye affected by EWM destruction from a bird housed in CL for 1 week post-lesion (**E**), and in an eye affected by EWM destruction from a bird housed in CL for 3 weeks post-lesion (**F**). Red cones (red R), principal cones (white P), green cones (green G), violet/blue cones (blue B), and rods (black R) are identifiable by the traits shown in Fig. 2A. Note that all photoreceptor types are identifiable in (**A** and **B**), but fewer violet/blue cones are evident in the eyes affected by EWM destruction from birds housed in normal DL (**C** and **D**). Violet/blue cones are also absent, and principal cones sparse or absent in eyes affected by EWM destruction from birds housed in CL (**E** and **F**). Note also that outer and inner segments in eyes affected by EWM destruction in the eye from the bird housed in CL for 3 weeks (**F**) showed additional abnormalities not seen in the one week eye, including shrinkage, vacuolation, and darkening of inner segments. By comparison to the retina shown in Fig. 2B and in images (**A** and **B**), the inner segments in (**C** and **D**) are largely normal in appearance, reflecting the more limited effects of EWM destruction on photoreceptors in birds housed in normal DL. All images are at the same magnification.

**Fig. 5.**
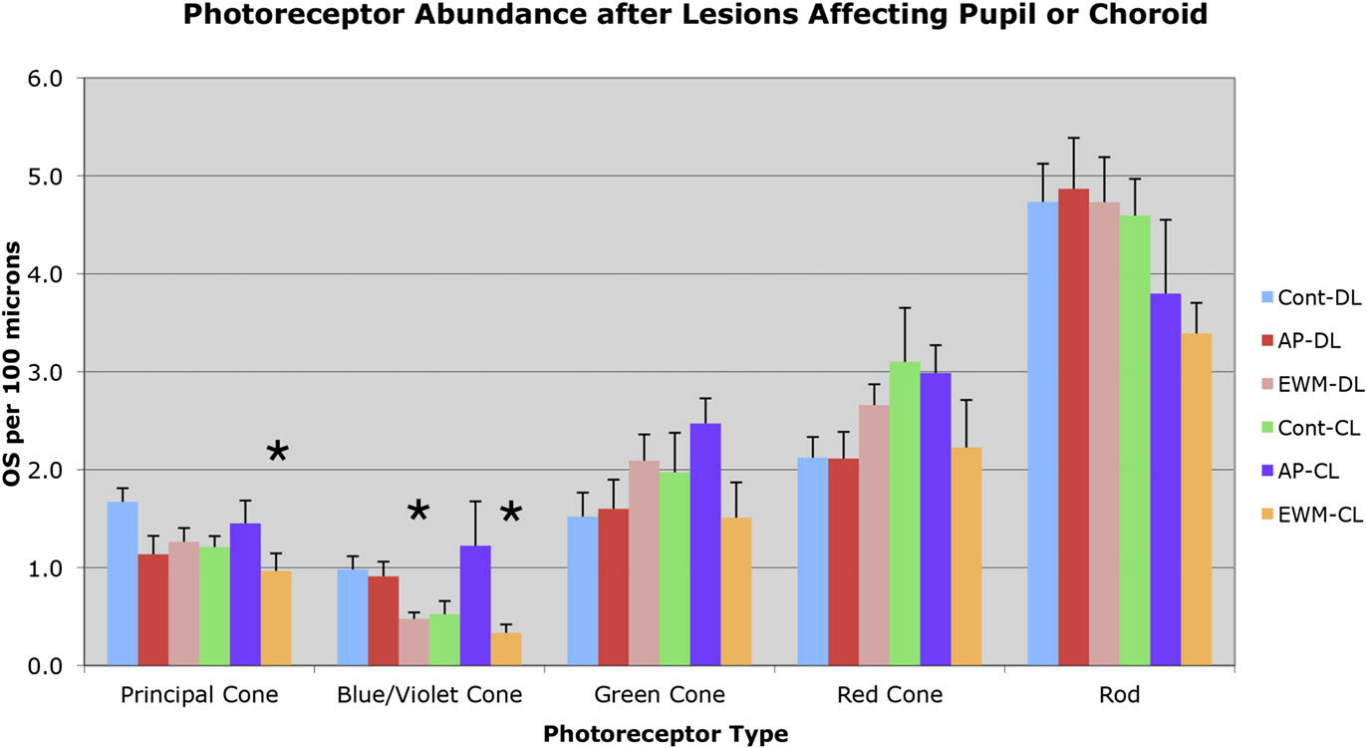
Graph illustrating the effects of the various manipulations performed on the abundance of outer segments of the different photoreceptor types in the superior central pigeon retina, per 100 *µ*m length of retina. Photoreceptor outer segments were identified and counted according to criteria and approaches described in the text. The greatest losses observed were for blue/violet cone outer segments following EWM destruction and housing in either DL or CL. Principal cone outer segment loss following EWM destruction and housing in CL was also seen. Error bars represent SEMs. Asterisks indicate significant differences from the control-DL group at the *P* < 0.0125 level.

No significant differences were found for principal cone outer segment abundance between the two control groups (control-DL and control-CL), nor between either of the AP groups and the control-DL group (Figs. 4 and 5). Thus, CL *per se* was not found to adversely affect principal cone outer segment abundance, since neither control-CL nor AP-CL principal cone outer segments showed significant loss. Although no significant change was seen in principal cone outer segment abundance in the EWM-DL group compared to the control-DL group, a significant 42.2% reduction was seen in principal cone outer segment abundance in the EWM-CL group compared to the control-DL group (*P* = 0.0096). To further evaluate the lesion effects on photoreceptor outer segment abundance, we performed regression analysis to determine if principal cone outer segments were undergoing survival-related decline that was perhaps not yet evident at the group level in some cases. We, however, found no significant correlation of principal cone outer segment loss with post-lesion survival for any of the DL or CL groups, indicating that loss of principal cones was only evidenced in the EWM-CL group and it was not markedly progressive over the 3 weeks of CL.

### Blue/violet cone outer segments

Blue/violet cones showed a significant outer segment loss following EWM destruction in both lighting conditions (Figs. 4 and 5). For example, blue/violet cone outer segment abundance in eyes affected by EWM destruction in birds housed in DL was 48.5% of that in the eyes of control-DL birds (*P* = 0.0095). A similar effect was seen for blue/violet cone outer segment abundance in EWM lesion-affected eyes in the CL-housed birds, whose blue/violet cone abundance was 34.0% of that in the control-DL eyes (*P* = 0.0070). The blue/violet cone outer segment abundance was, however, not significantly less in AP experimental eyes than that in control-DL eyes for either lighting group (Fig. 5). The blue/violet cone outer segment loss was significantly correlated with survival time in EWM experimental eyes in birds housed in CL (*r* = −0.867), but not in any of the other groups, indicating it was progressive in the EWM-CL group.

### Green cone outer segments

Green cones did not show any reduction in outer segment abundance in AP or EWM lesion groups under either the DL or CL conditions, compared to the control-DL eyes (Figs. 4 and 5). We also found no significant correlations of green cone outer segment abundance with post-lesion survival in any of the DL groups, or for the control-CL and AP-CL groups. Although green cone outer segment loss *per se* was not significant in eyes affected by EWM destruction in CL birds compared to control-DL eyes at a group level, green cone outer segment abundance in the EWM-CL group tended to be less than in the other CL groups. Moreover, green cone outer segment abundance in EWM-CL trended toward a significant inverse correlation with post-lesion survival (*r* = −0.817), which however did not achieve the 0.8433 required for significance. These results suggest that some decline in green cones may have been in progress in EWM-CL eyes.

### Red cone outer segments

Red cones did not show any reduction in outer segment abundance in AP or EWM lesion groups under either the DL or CL conditions, compared to the control-DL eyes (Figs. 4 and 5). We also found no significant correlations with post-lesion survival in any of the groups for red cone outer segment abundance.

### Rod outer segments

No significant differences were seen in the comparisons between AP-DL, EWM-DL, and control-CL eyes to control-DL eyes for rod outer segment abundance (Figs. 4 and 5). Although rod outer segment abundance in EWM-CL eyes was about 70% of that in the control-DL eyes, the difference was not significant (*P* = 0.0816), although it trended in that direction. The rod outer segment abundance in the AP-CL group also appeared to trend toward reduction (Fig. 5), but the difference was not significant compared to the control-DL eyes (*P* = 0.2731). We found no significant correlations with post-lesion survival in any of the DL or CL groups for rod outer segment abundance, indicating no prominent trend toward progressive loss of rod outer segments over the survival times used. Although rod outer segments did not yet show significant loss after three weeks of CL, rod photoreceptor pathology in the form of darkened and degenerating inner segments was seen (Fig. 4).

## Discussion

Our findings show that EWM lesions that disrupt parasympathetic control of ChBF adversely affect short wavelength-sensitive blue/violet cones and principal cones, and this effect is exacerbated by CL. These photoreceptor types have been shown in prior studies in mammals and birds to be preferentially vulnerable as well to light, hypoxia, and aging (Greenstein et al., 1989; Hodos et al., 1991; Fite et al., 1993; Machida, 1994; Curcio, 2001; Algvere et al., 2006; Organisciak & Vaughn, 2010; Hovis et al., 2012). Our results and their implications are discussed in more detail below.

### Prior studies in birds

The circuit from the retina to the EWM *via* the vSCN (Fig. 1) mediates increases in ChBF in response to retinal illumination (Fitzgerald et al., 1990*a*,*b*, 1996; Shih et al., 1993). Lesions of EWM or sectioning of the choroidal nerve from CG to the choroid reduces basal ChBF and prevent light-mediated reflexive increases in ChBF (Shih et al., 1994; Fitzgerald et al., 1996). Our prior studies in pigeons show that the ChBF insufficiency caused by EWM destruction in young adult pigeons causes progressive increases in Müller cell GFAP, so that by one year after lesion GFAP immunolabeling of Müller cells extends throughout the entire depth and extent of the ipsilateral retina (Fitzgerald et al., 1990*a*; Kimble et al., 2006). GFAP upregulation in retinal Müller cells is a well-known correlate of retinal injury, stress, or disease (Bignami & Dahl, 1979; Eisenfeld et al., 1984; Burns & Robles, 1990; Osborne et al., 1991; Humphrey et al., 1993; Sarthy & Egal, 1995; Tanihara et al., 1997; Chen & Weber, 2002), and its occurrence after an EWM lesion thus implies retinal injury. Consistent with this interpretation, increases in GFAP expression have also been found following transient choroidal and/or retinal ischemia (Gay et al., 1964; Hayreh & Weingeist, 1980; Osborne et al., 1991; Tanihara et al., 1997; Kim et al., 1998). We have further shown that the retinal abnormalities caused by an EWM lesion in young adult pigeons housed in DL result in reduced visual acuity by about one year post-lesion (Hodos et al., 1998). Our present studies suggest that blue/violet and principal cone loss may have contributed to these acuity deficits. Given that loss of principal cones and accessory cones occurs together with age in pigeons (Hodos et al., 1991), it seems likely that EWM destruction may have affected accessory cones as well.

In our prior study reporting retinal GFAP increases after EWM destruction (Kimble et al., 2006), we saw no significant GFAP increase over the two months after lesions that disrupted the PLR without affecting ChBF control (i.e., by destruction of either AP or EWLcl). In the present study as well, PLR disruption alone had no significant effect on photoreceptors in either normal DL or in CL. The prominent outer segment loss seen in EW lesion birds thus appeared to require disruption of ChBF regulation by EWM. Our observation that retinal pathology was insignificant after lesions that disrupt pupil alone is consistent with our acuity data, showing that acuity is unimpaired in pigeons one year after pupil light reflex elimination by means of AP destruction (Hodos et al., 1998). It remains possible that longer post-lesion survivals would reveal an impact on photoreceptor health after pupil light reflex disruption alone, perhaps progressing to prominent outer segment loss. Studies in humans with diseases that impair pupil constriction are consistent with this possibility (Laor & Korczyn, 1978; Gräf & Jungherr, 2002).

### Light damage

The damaging effects of transient exposure to extremely bright light, or sustained exposure to moderately bright or normal light, on photoreceptors have been demonstrated in a variety of species (Marshall et al., 1972; Fite et al., 1993; Machida, 1994; Pérez & Perentes, 1994; Thomson et al., 2002; Algvere et al., 2006; Organisciak & Vaughn, 2010; Thomas et al., 2012). Rods are particularly vulnerable to light damage, especially in nocturnal species (Cicerone, 1976; Tanito et al., 2007; Organisciak & Vaughn, 2010; Okano et al., 2012). By contrast, rod vulnerability to CL appears to be lower in pigeons than in nocturnal rodents, as true for other diurnal species in general, since no significant rod loss was seen for any of the bird groups housed in CL for up to three weeks. A trend in that direction was, however, observed for the EWM-CL eyes. We also observed low cone vulnerability in control eyes or AP-lesion affected eyes from birds housed in CL. By contrast, Marshall et al. (1972) reported cone outer segment loss in pigeons that were exposed to 1000 lux light for at least 24 h. It is possible then that more sustained and/or brighter lighting than we used (400 lux for up to 3 weeks) is needed to cause cone outer segment loss in normal or AP lesion pigeon eyes.

Our prior study (Kimble et al., 2006) and the current one indicate that the adverse effects of EWM circuit destruction on the retina are exacerbated by light, suggesting that ChBF regulation by the EWM circuit depicted in Fig. 1 acts to mitigate light damage to photoreceptors. Photoreceptors show high metabolic activity in response to flickering light (Bill & Sperber, 1990), and ChBF in pigeons has been shown to increase in response to flickering light (Shih et al., 1997). It may be then that the unrelenting photoreceptor metabolic demand from a constantly changing retinal image in an illuminated cage, especially one that is constantly illuminated, may require sustained high ChBF. Moreover, inner retinal demand may exacerbate outer retinal vascular insufficiency, since the choroid is the sole vascular supply for the entire depth of the avian retina (Bill & Sperber, 1990). As a result, the outer retina in pigeons following EWM destruction would experience ongoing choroidal insufficiency, causing hypoxic-ischemic injury (Gaudric et al., 1982; Yu & Cringle, 2001). Hypoxic insults to outer retina and/or diminished ChBF are known to heighten Müller cell GFAP expression (Penn et al., 1988; Canady et al., 1990), and cause RPE and photoreceptor dysfunction and loss (Linsenmeier et al., 1983; Linsenmeier & Steinberg, 1984; Yancey & Linsenmeier, 1988; Ciulla et al., 2001; Johnson et al., 2005; Yu & Cringle, 2005).

### Differential photoreceptor vulnerability

In the present study, we found that short wavelength cones and principal cones were most vulnerable to a disturbance in parasympathetic regulation of ChBF by the EWM circuit. Prior studies have also found these to be among the most vulnerable of the photoreceptor cell types. For example, short wavelength cones in diurnal species, including avian species, have been reported to be vulnerable to: (1) constant or bright light (Sperling et al., 1980; Sykes et al., 1981; Sperling, 1986; Machida, 1994); (2) hypoxia (Smith et al., 1976; Algvere et al., 2006; Connolly et al., 2008; Hovis et al., 2012); and (3) aging (Eisner et al., 1986; Haegerstrom-Portnoy, 1988; Gao & Hollyfield, 1992; Curcio et al., 1993; Gray et al., 1995; Algvere et al., 2006; Okano et al., 2012). Blue cone vulnerability and loss in humans have also been observed in diabetic retinopathy, glaucoma, and retinitis pigmentosa (Greenstein et al., 1989). The particular vulnerability of short wavelength cones may involve the greater photic energy of short wavelength light or the greater metabolic requirements of blue cones (Penn & Anderson, 1992; Young, 1992; Algvere et al., 2006; Connolly et al., 2008). As EWM destruction would render the affected eye hypoxic-ischemic, it is not surprising that this cone type should be vulnerable following disabling of parasympathetic control of ChBF. Accessory and principal cones in pigeons are preferentially vulnerable to age-related loss (Hodos et al., 1991; Fitzgerald et al., 2001). Hodos et al. (1991) suggested that age-related accessory and principal cone loss might reflect the cumulative effects over a lifetime of vulnerability to light damage, because they lack the protection provided by a colored oil droplet and are highly light sensitive (Mariani & Leure-Dupree, 1978; Bowmaker et al., 1997). Moreover, their coupling to one another *via* gap junctions (Smith et al., 1985) may cause them to exacerbate the light damage to each other.

In the present study, we also observed a suggestion of rod vulnerability in EWM-lesioned birds housed in CL, in that we observed a trend toward rod outer segment loss and we also observed darkened and degenerating rod inner segments. Rods are known to be particularly vulnerable to light damage, especially in nocturnal species (Cicerone, 1976; Tanito et al., 2007; Organisciak & Vaughn, 2010; Okano et al., 2012). Their particular vulnerability to light damage has been attributed to their light sensitivity, which is especially acute in nocturnal species. Light-induced generation of reactive oxygen species has been thought to be a major contributor to rod phototoxicity, with subsequent peroxidation of outer segment lipids (Organisciak & Vaughn, 2010). It may be that diminished parasympathetic control of ChBF heightens sensitivity of rods to light damage by impairing energy-dependent anti-oxidant defenses (Organisciak & Vaughn, 2010).

### Disease implications

A link between deficient parasympathetic ChBF regulation and outer retinal pathology and dysfunction has been shown in our prior studies of aging pigeons (Fitzgerald et al., 2001). In humans, parasympathetic innervation of the choroid by the pterygopalatine ganglion is diminished in normal aging, as is basal ChBF (Grunwald et al., 1998; Ito et al., 2001; Jablonski et al., 2007). Moreover, age appears to impair the adaptive stabilization of ChBF by the parasympathetic nervous system in response to systemic blood pressure fluctuations (Reiner et al., 2011), which appears to be a major function of the pterygopalatine parasympathetic input to the choroid (Cuthbertson et al., 2003; Li et al., 2010; Reiner et al., 2003, 2010). The present results raise the possibility that age-related impairments in adaptive vasodilatory ChBF control may contribute to the age-related waste accumulation in and along Bruch’s membrane and photoreceptor loss observed in humans (Potts, 1966; Tso, 1988). Yet more profound declines in ChBF, as well as in its baroregulation, occur in dry and wet age-related macular degeneration (AMD), with the ChBF declines increasing in severity with AMD severity, and predicting the development of neovascularization (Friedman et al., 1995; Ciulla et al., 2001; Grunwald et al., 2005; Pournaras et al., 2006; Metelitsina et al., 2008; Feigl, 2009). In individuals with pro-AMD genetic predispositions in the alternate complement cascade or in lipid metabolism, such waste accumulation may trigger an inflammatory response that damages RPE and photoreceptors (Winkler et al., 1999; Hageman et al., 2008; Feigl, 2009). Thus, deficient vasodilatory ChBF regulation may play a role in age-related retinal decline and be a risk factor for AMD (Hageman et al., 2008).
